# Complete Genome Sequence of the *Bacillus pumilus* Phage Leo2

**DOI:** 10.1128/genomeA.00066-18

**Published:** 2018-02-15

**Authors:** Sondos Badran, Nathanael Morales, Phillip Schick, Brandon Jacoby, William Villella, Todd Lorenz

**Affiliations:** a Biology Department, University of La Verne, La Verne, California, USA; b Microbiology, Immunology, and Molecular Genetics, University of California, Los Angeles, Los Angeles, California, USA

## Abstract

*Bacillus* spp. are ubiquitous Gram-positive microbes with many ecological and symbiotic interactions and can be pathogens. Phage Leo2 was found to infect a *Bacillus pumilus* strain isolated from soil. The sequence of phage Leo2 revealed 74 genes; 31% of the genes have associated functions, and 67% of coding regions are unidentified open reading frames.

## GENOME ANNOUNCEMENT

*Bacillus* spp. are well-documented bacteria, yet only ∼100 *Bacillus* phage genomes have been sequenced (NCBI Genome database search, 19 January 2018). *B. pumilus* phages have fewer than 15 sequenced genomes (1–6). The first *B. pumilus* phage that was isolated differs from other *Bacillus* phages in burst size and latent period (7). Since then, several studies have isolated and characterized *B. pumilus* phages without sequencing (8–12). Recent studies have reduced the knowledge gap in *B. pumilus* phage genetics by finding, clustering, and annotating several phages (1–6).

Phage Leo2 was isolated from rhizosphere soil of a *Buxus* sp. at the University of La Verne. The phage was characterized morphologically using transmission electron microscopy as a *Siphoviridae*, with a noncontractile tail and an icosahedral head. However, the phage has novel morphological characteristics (capsid and tail length) compared to those of the other Andromeda-like phages, which are a group of similar *Siphoviridae* phages that infect *B. pumilus* (1). The capsid length is 70.7 nm ± 1 nm, with a width of 68.9 nm ± 2 nm, and the tail length measures 128 nm ± 6 nm. Phage Leo2 has a larger head length and width than those of other *B. pumilus* phages. Phage Leo2 has a genome length (48,589 bp) that is comparable to that of other Andromeda-like phages and a GC content of 42.09%.

We sequenced phage Leo2 with 454 pyrosequencing at UCLA's GenoSeq facility. Raw sequence data were assembled using Newbler Assembler with 154-fold coverage. Final assembly and alignment of the genome were accomplished using Consed (13). The phage Leo2 genome was annotated using DNA Master (14). Putative gene functions were analyzed using HHPred, BLAST (Basic Local Alignment Search Tool), and Pfam (15). Phage Leo2 has 74 annotated genes and a genome arrangement similar to that of Andromeda-like phages. Based on whole-genome BLAST analysis, phage Leo2 was closely related to phages Gemini and Taylor, displaying 92% and 85% query coverages and 84% and 89% identities, respectively, suggesting that it is a member of the BpA cluster of Andromeda-like phages (1).

Similarities to the genomic arrangement of the Andromeda-like phages are also found, starting with eight small genes observed in many of the BpA genomes. The terminase gene (Leo2_9) begins the structural cassette, which includes the portal protein (Leo2_10), minor head protein (Leo2_11), scaffold protein (Leo2_14), major capsid protein (Leo2_16), phage virion morphogenesis protein (Leo2_19), tape measure protein (Leo2_25), and tail spike protein (Leo2_28), and is followed by an *N*-acetylmuramoyl-l-alanine amidase (Leo2_32). Other genes of known function consist of bacterial origin and uncharacterized open reading frames (ORFs). The 23 putative gene functions are organized into five categories. The first category of putative gene functions are associated with virion assembly or structural genes, which are conserved and include the terminase, portal protein, minor head protein, scaffold protein, major capsid protein, virion morphogenesis protein, TMP, and tail spike protein. Six of the genes are DNA replication genes, gp38 (DNA primase), gp41 (DNA polymerase III), gp65 (replication and relaxation proteins), gp37 (DNA helicase), and gp64 (Ftsk/SpoIIIE ATPase). There were two genes recognized for regulation of transcription, gp40 (transcriptional regulators) and gp44 (RNA polymerase sigma-70 factor). Four genes were identified that are involved in bacterial metabolic processes, including gp52 and gp54 (kinases), gp32 (amidase), and gp46 (synthase). Lastly, three DNA recombination genes were found in phage Leo2, including gp42 (RecB-like protein nuclease), gp43, and gp71.

### Accession number(s).

The genomic data have been entered into GenBank and are recorded under the accession number KU836751.
